# Helminth diversity in *Pimelodus blochii* Valenciennes, 1840 (Osteichthyes: Pimelodidae) in two Amazon Rivers

**DOI:** 10.1007/s00436-020-06906-x

**Published:** 2020-10-12

**Authors:** Pedro Hercílio de Oliveira Cavalcante, Maralina Torres da Silva, Aldenice de Nazaré Silva Pereira, Rosana Gentile, Cláudia Portes Santos

**Affiliations:** 1 Instituto Federal do Acre, Campus Rio Branco, Rio Branco, AC Brazil; 2 Programa de Pós-Graduação em Biodiversidade e Saúde, Instituto Oswaldo Cruz, Fiocruz, Rio de Janeiro, Brazil; 3 Instituto Federal do Pará, Campus Abaetetuba, Abaetetuba, PA Brazil; 4 Laboratório de Biologia e Parasitologia de Mamíferos Silvestres Reservatórios, Instituto Oswaldo Cruz, Fiocruz, Rio de Janeiro, RJ Brazil; 5 Laboratório de Avaliação e Promoção da Saúde Ambiental, Instituto Oswaldo Cruz, Fiocruz, Av. Brasil 4365, Rio de Janeiro, RJ 21040-360 Brazil

**Keywords:** Host-parasite relationship, Freshwater fish, Xapuri River, Acre River, Western Amazonia

## Abstract

Structure of the helminth community and analyses of helminth population parameters of *Pimelodus blochii* collected in the Xapuri River in comparison with those in the Acre River were evaluated. Eight adult helminth species were found parasitizing *P. blochii* in the Acre River: the nematodes *Orientatractis moraveci*, *Rondonia rondoni*, *Philometroides acreanensis*, *Cucullanus* (*Cucculanus*) *pinai pinai*, *Procamallanus* (*Spirocamallanus*) *pimelodus*, *Rhadochona acuminata*, and *Brasilnema* sp., and the trematode *Dadaytrema oxycephala.* For Xapuri’s fishes, nine helminth species were found: the nematodes *O. moraveci*, *R. rondoni*, *C.* (*C.*) *pinai pinai*, *Procamallanus (Spirocamallanus) rarus*, *P.* (*S*.) *pimelodus*, *R. acuminata*, *Brasilnema* sp., and Cystidicolidae gen. sp., and the trematode *D. oxycephala.* Nematode and Acanthocephala larvae were also reported. Helminth abundance, prevalence, and diversity were influenced by seasonality and locality (river). The helminth parasites from Acre’s fishes formed a subset of the helminth community of the Xapuri’s. The results indicate an influence of the environmental characteristics of the rivers on the helminth community structure and diversity. This is the first study of the parasite community of *P. blochii* in the Xapuri River. The paretheses of (Spirocamallanus) and (S.) should not be in italics all along the text and tables.

## Introduction

The constant changes in the natural environments caused by the anthropic activities may alter the species composition as well as the structure of the biological communities. This process may favor certain species to the detriment of others, thus affecting biodiversity. In addition, disturbances in the environment may also favor the occurrence of parasites and alter their distributions (Combes 2001), influencing the host-parasite interactions in natural ecosystems. The dumping of human sewage from cities in rivers is one of the most important causes of disturbance in freshwater environments (Wen et al. 2017). Fish parasites may be good indicators of water quality, especially helminths whose free-living stages are more sensitive to environmental changes, decreasing their population sizes in polluted environments (Mackenzie et al. 1995; Palm 2011).

*Pimelodus blochii* Valenciennes, 1840 is an endemic freshwater fish from the Neotropical region, occurring in the Amazon, Paraná, Orinoco, and Guiana basins (Lundberg and Littmann 2003; Eschmeyer and Fong 2014). This species is characterized by living in groups, commonly found under logs in the benthic environments (Le Bail et al. 2000 apud Lundberg and Littmann 2003). Its feeding habit is diverse, what suggests a large trophic adaptation varying according to the availability of the prey (López-Casas and Jiménez-Segura 2007). This species feeds on macroinvertebrates and fruits but may also act as a detritivore (Lundberg and Littmann 2003).

In the north of Brazil, this fish is an appreciated food item. The commercial exploitation of this species has been evaluated (Maciel et al. 2014) due to its high abundance and facility use in the filleting process. The use of this species in human feeding arouses the interest in studying its parasites, especially in investigating the occurrence of parasites of human interest. Concerning helminth parasites, most of the studies carried out for this host were species lists (Kohn et al. 2007; Luque and Tavares 2007; Luque et al. 2011; Martins 2018) or species descriptions (Gil de Pertierra 2004; Mendoza-Palmero and Scholz 2011; Orélis-Ribeiro and Bullard 2015; Cavalcante et al. 2017; Cavalcante et al. 2018; Negreiros et al. 2019a; Negreiros et al. 2020). There are only two studies which investigated the helminth community structure of *P. blochii* (Negreiros et al. 2018, 2019b). The former compared the parasitic fauna in the Acre and Iaco Rivers in the Amazon region, reporting 22 taxa with a dissimilar parasite community structure between rivers; the latter evaluated the temporal changes in the community structure also in the Iaco River for 5 years.

Considering the scarce information concerning the ecological aspects of the parasitism of this fish, the aim of this study was to describe the helminth species composition and to analyze the parasitological parameters and the community structure of the helminths of the catfish *P. blochii* in two Amazon rivers with different levels of anthropic pollution, Acre and Xapuri Rivers, state of Acre, Brazil. We also evaluated the occurrence of helminths with potential risk to human health. We suggest that helminth species composition and diversity vary between rivers in response to their local characteristics, and between seasons, due to the rainfall pattern observed in the region.

## Material and methods

### Study area

The study area is located in the state of Acre in the Amazon biome, which vegetation is characterized by tropical rainforests. The state of Acre is one of the most preserved Brazilian states, with 11.3% of deforested area. The region presents tropical rainforest climate (Af), according to the Köppen classification, characterized by high temperatures, varying from 22 to 26 °C, and high rainfall levels, varying from 1600 to 2500 mm annually (Ayoade 1986). The dry season, which is during the winter, is very short, and rainfall is normally heavy throughout the year.

**Fig. 1:**
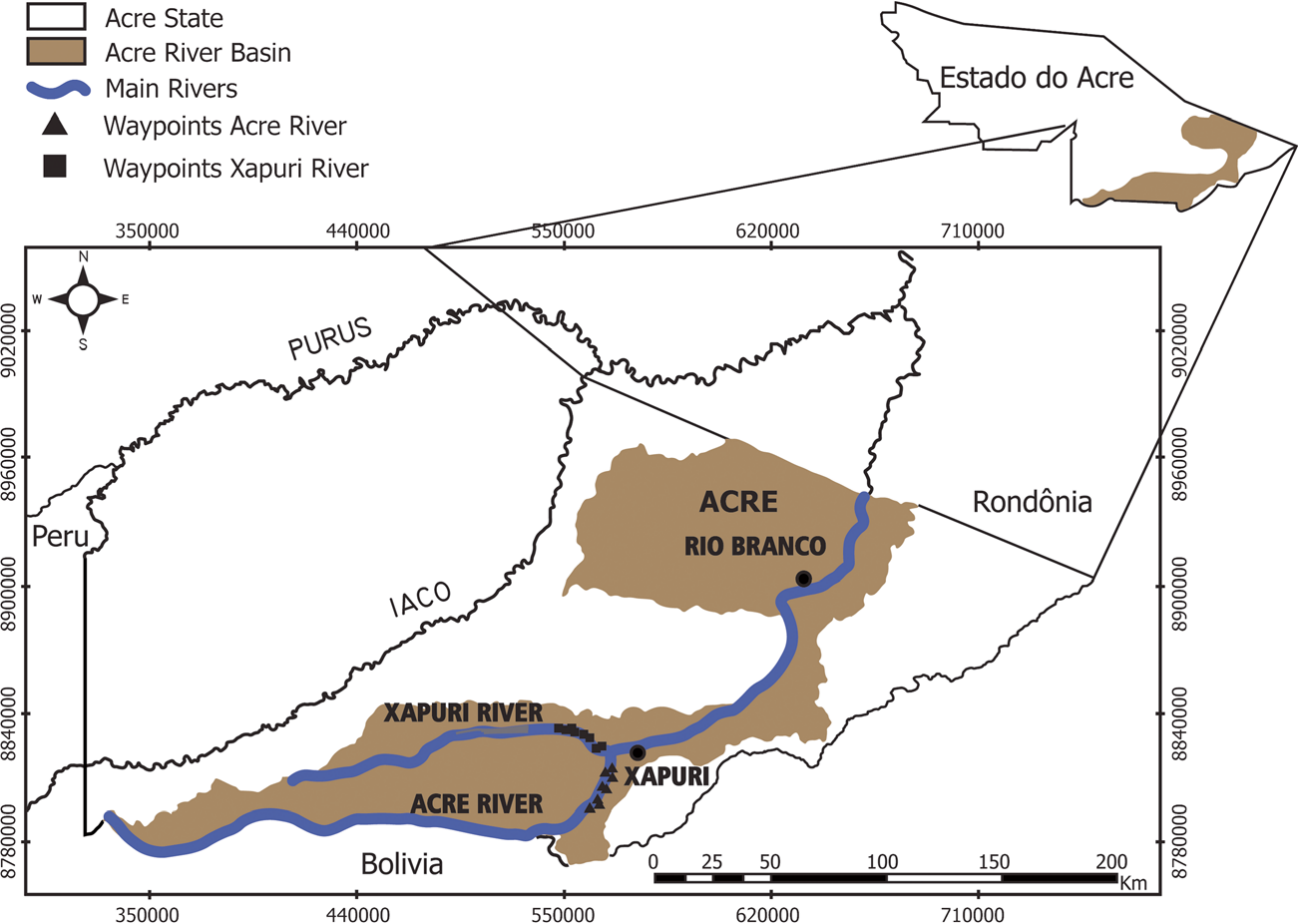
Study area of the helminth fauna of *Pimelodus blochii*, State of Acre, Brazil, indicating the location of Acre, Xapuri, Iaco, and Purus Rivers in Brazil. Adapted from Macêdo et al. (2013)

Fish samplings were carried out in Acre (10°39′40″S, 68°30′19″W) and Xapuri Rivers (10°38′20″S, 68°32′08″W), in the municipality of Xapuri (Fig. 1). The Acre River has its headspring in Peru and is an affluent of the Purus River, which in turn is an affluent of the Amazon River. It extends about 1190 km crossing the state of Acre, where it receives the human sewage from several cities through which it passes, including the state’s capital, Rio Branco (Furtado and Lopes 2018). The Xapuri River is the main affluent of the Acre River. Its headspring is in the Extractive Reserve Chico Mendes, a preserved area of 970,000 ha with sustainable economic activities. The river crosses the reserve until it debouches to the Acre River.

During the short dry season, the level of the rivers becomes shallow, forming pockets of water at certain points, where fish populations are concentrated. During this season, the rivers can be easily crossed by foot. In the peak of the rainy season, the level of the rivers increases, allowing navigation. The rivers reach a depth of more than 10 m, with the vegetation in their banks being dragged by the force of the river flow. These effects are more accentuated for the Acre River, due to the existence of different cities in its surroundings. The less protected banks and waters receiving the sewage from the houses make this river more impacted than the Xapuri one. The latter is more preserved, as it does not pass along cities, only by local riverside communities with approximately 3000 inhabitants (Acre 2010), receiving much less anthropogenic impacts.

### Sampling methods

The fishes were collected by local fishermen using cast nets. From 2013 to 2015, 240 specimens of *P. blochii* were analyzed for helminth presence. For each river, 120 specimens were collected, 60 in the wet season (summer) and 60 in the dry season (winter). Fishes were collected in both rivers at the same periods concerning seasons and years in order to enable comparisons. This research was authorized by the Chico Mendes Institute for Biodiversity Conservation (license ICMBio No. 43450-1), according to the guidelines of the Brazilian College of Animal Experimentation (COBEA).

The specimens of fish were packed in ice and taken to the laboratory where they were weighed and measured. The external surface of the fishes was analyzed and the internal organs were separated in Petri dishes with 0.7% physiological solution and analyzed using a stereoscopic microscope. The nematodes and acanthocephalans collected were fixed in 70% alcohol or 4% heated formalin. The trematodes were fixed in 70% alcohol under slight compression. The nematodes were clarified in glycerine and the trematodes were stained with Meyer’s paracarmine, cleared in clove oil, and mounted in Canada Balsam. The helminths were counted, measured, and identified to the highest taxonomic level as possible, according to Moravec (1998), Thatcher (2006), Luque et al. (2011), and Cavalcante et al. (2017, 2018).

### Data analysis

Mean abundance, mean intensity, and prevalence were calculated for each helminth species, regarding the river and the season, according to Bush et al. (1997). Mean abundance was considered as the total number of helminths of a species divided by the number of hosts analyzed. The mean intensity was the total number of helminths of a certain species divided by the number of animals infected by this species. The prevalence was the ratio between the number of infected animals and the total number of animals analyzed. Aggregation indices were calculated for each helminth species whose prevalence was higher than 10% using the variance-to-mean ratio of the parasite abundance.

Abundances were compared in relation to river and season only for the four most abundant species, considering each species separately, using generalized linear models (GLM) following a Gaussian distribution. The best models were chosen using the corrected Akaike information criterion (AICc), in which the plausible models presented ∆AICc ≤ 2. Prevalence rates were compared between rivers and between seasons within each river using the chi-square contingency test.

We used sample-based rarefaction curves (Mao Tau method) in order to compare the species richness between the helminth communities of the two rivers. This analysis was carried out using the absolute abundance of each species sampled considering each fish as a sampling unit.

Mean richness of the helminths was measured for each river and season as the sum of the species found in each individual host divided by the number of hosts analyzed. Total species richness was measured as the number of helminth species found. Expected richness was estimated using the Chao-1 estimator. This index was used in order to evaluate the adequacy of the sample size in relation to species richness, when compared with the observed values. Helminth species diversity was calculated using the Shannon index (*H*) for each river and season and compared by *t* test. Equitability was calculated using Pielou’s J index. Community species dominance was evaluated by the Berger-Parker index. Dissimilarities in the helminth communities between the two rivers were investigated using the beta-diversity and its components: beta-diversity nestedness (*β*nes) and beta-diversity turnover (*β*turn), in order to investigate whether diversity is driven by loss (*β*nes component) or replacement (*β*turn component) of helminth species in the communities. This analysis was done with abundance data using the Bray-Curtis dissimilarity index and the beta-diversity method of Baselga (2013).

Importance indices of helminth species were calculated according to Thul et al. (1985) for each river. Each helminth species was classified in the community as dominant species (*I* ≥ 1.0), co-dominant (0.01 ≤ *I* < 1.0), subordinate (0 < *I* < 0.01), or unsuccessful pioneer (*I* = 0).

All the analyses were carried out only for adult helminth species. A significance level of 5% was used in the analyses. Univariate tests, rarefaction curves, and diversity and richness estimates were performed using the Past software, version 3:09 (Hammer et al. 2001). The GLM analyses were performed using the *vegan* package (Oksanen et al. 2018) and the beta-diversity analysis using the *betapart* package (Baselga et al. 2017), both in R Core Team (2018).

## Results

In total, 2676 adult helminths were recovered, 595 from fishes of the Acre River, where 37.5% were infected, and 2081 of the Xapuri River, where 55% were infected. In the Acre River, fishes presented total body length ranging from 8.8 to 25.7 cm with an average ± standard deviation of 18.55 ± 2.83 and body mass between 15.7 and 160 g with an average of 50.68 ± 23.76. In the Xapuri River, fishes presented total length varying between 13.7 and 23.2 cm with an average of 18.75 ± 1.77 and body mass between 20.5 and 92.7 g with an average of 46.26 ± 13.88.

Eight helminth morphospecies were found in the Acre River, considering the adult specimens: the nematodes *Orientatractis moraveci* Cavalcante, Silva, Santos, Chagas-Moutinho & Santos, 2017; *Rondonia rondoni* Travassos, 1920; *Philometroides acreanensis* Cavalcante, Moravec & Santos, 2018; *Cucullanus* (*Cucculanus*) *pinai pinai* Travassos, Artigas & Pereira, 1928; *Procamallanus *(*Spirocamallanus*)* pimelodus* Pinto, Fábio, Noronha & Rolas, 1974; *Rhadochona acuminata* (Molin, 1860), and *Brasilnema* sp., and the trematode *Dadaytrema oxycephala* (Diesing, 1836)*.* For Xapuri’s fishes, nine helminth morphospecies were found: the nematodes *O. moraveci*, *R. rondoni*, *C.* (*C.*) *pinai*, *Procamallanus *(*Spirocamallanus*)* rarus* Travassos, Artigas & Pereira, 1928, *P. *(*S.*)* pimelodus*, *R. acuminata*, *Brasilnema* sp. and Cystidicolidae gen. sp., and the trematode *D. oxycephala.* We also found larvae of the nematodes *Hysterothylacium* sp. (14 specimens in 11 fishes), *Anisakis* sp. (5 specimens in 5 fishes), Nematoda gen. sp. (55 specimens in 37 fishes), and of Acanthocephala gen. sp. (121 specimens in 31 fishes) in fishes from both rivers. Larvae of the nematode *Contracaecum* sp. (2 specimens in 2 fishes) were found only in Xapuri’s fishes.

### Helminth populations

The most abundant and prevalent helminth species were *O. moraveci*, *P.* (*S.*) *pimelodus*, and *R. rondoni* in both rivers (Tables 1 and 2). In Xapuri River, *Brasilnema* sp. also had high values of prevalence and abundance. All species which prevalences were higher than 10% showed an aggregated index of dispersion (Tables 1 and 2).

**Table 1:** Mean abundance, mean intensity (± SD), and prevalence (± 95% CI) for the helminth fauna of *Pimelodus blochii* in the Acre River, State of Acre, Brazil. (-) indicates that the parameter was not estimated

Acre river								
Parameters	Species							
Total	*Orientatractis moraveci*	*Rondonia rondoni*	*Philometroidesacreanensis*	*Cucullanus pinai pinai*	*Procamallanus *(*S.*) *pimelodus*	*Dadaytrema oxycephala*	*Rhadochonaacuminata*	*Brasilnema* sp*.*
Prevalence	14.17 (0.09)	7.50 (0.01)	0.83 (0.0)	5.00 (0.0)	12.50 (0.02)	5.00 (0.0)	2.50 (0.0)	3.33 (0.0)
Mean abundance	3.51 ± 6.17	0.45 ± 0.83	0.02 ± 0.03	0.08 ± 0.16	0.72 ± 1.25	0.08 ± 0.14	0.03 ± 0.05	0.08 ± 0.16
Mean intensity	24.76 ± 25.08	6.00 ± 5.33	2.00 ± 0	1.67 ± 0.89	5.73 ± 5.56	1.50 ± 0.50	1.00 ± 0	2.50 ± 0.75
Total abundance	421	54	2	10	86	9	3	10
Aggregation index	76.54	12.66	-	-	10.49	-	-	-
Parameters	Species							
Dry season	*Orientatractis moraveci*	*Rondonia rondoni*	*Philometroides acreanensis*	*Cucullanus pinai pinai*	*Procamallanus *(*S.*) *pimelodus*	*Dadaytrema oxycephala*	*Rhadochonaacuminata*	
Prevalence	10.83 (0.12)	4.17 (0.03)	0.83 (0.0)	2.50 (0.0)	4.17 (0.03)	2.50 (0.0)	1.67 (0.0)	
Mean abundance	2.20 ± 7.21	0.38 ± 1.41	0.02 ± 0.07	0.06 ± 0.22	0.37 ± 1.34	0.03 ± 0.13	0.02 ± 0.66	
Mean intensity	20.31 ± 14.87	9.20 ± 3.32	2.00 ± 0.26	2.33 ± 0.56	8.80 ± 3.20	1.33 ± 0.31	1.00 ± 0.18	
Total abundance	264	46	2	7	44	4	2	
Parameters	Species							
Rainy season	*Orientatractis moraveci*	*Rondonia rondoni*	*Cucullanus pinai pinai*	*Procamallanus *(*S.*) *pimelodus*	*Dadaytrema oxycephala*	*Rhadochona acuminata*	*Brasilnema* sp*.*	
Prevalence	3.33 (0.14)	3.33 (0.0)	2.50 (0.0)	8.33 (0.02)	2.50 (0.0)	0.83 (0.0)	3.33 (0.01)	
Mean abundance	1.31 ± 4.94	0.07 ± 0.25	0.03 ± 0.09	0.35 ± 1.17	0.04 ± 0.16	0.01 ± 0.03	0.08 ± 0.31	
Mean intensity	39.25 ± 17.85	2.00 ± 0.54	1.00 ± 0.22	4.20 ± 2.21	1.67 ± 0.38	1.00 ± 0.13	2.50 ± 0.67	
Total abundance	157	8	3	42	5	1	10

**Table 2:** Mean abundance, mean intensity (± SD), and prevalence (± 95% CI) for the helminth fauna of *Pimelodus blochii* in the Xapuri River, State of Acre, Brazil. (-) indicates that the parameter was not estimated

Xapuri river									
Parameters	Species								
Total	*Orientatractis moraveci*	*Rondonia rondoni*	*Cucullanus pinai pinai*	*Procamallanus *(*S.*)* rarus*	*Procamallanus *(*S.*)* pimelodus*	*Dadaytrema oxycephala*	*Brasilnema* sp*.*	Cystidicolidae gen. sp.	*Rhadochona acuminata*
Prevalence	23.33 (0.017)	19.17 (0.17)	1.67 (0.0)	0.83 (0.02)	35.83	5.83 (0.0)	15.00 (0.05)	1.67 (0.0)	2.50 (0.0)
Mean abundance	6.91 ± 11.75	5.82 ± 10.24	0.08 ± 0.16	0.01 ± 0.02	1.79 ± 2.43	0.11 ± 0.20	2.58 ± 4.51	0.02 ± 0.03	0.03 ± 0.06
Mean intensity	29.61 ± 32.22	30.35 ± 39.82	5.00 ± 3.00	1.00 ± 0	5.00 ± 0	1.86 ± 1.22	17.17 ± 1.57	1.00 ± 0	1.33 ± 0.44
Total abundance	829	698	10	1	215	13	309	2	4
Aggregation index	121.60	153.21	-	-	9.22	-	34.37	-	-
Parameters	Species								
Dry season	*Orientatractis moraveci*	*Rondonia rondoni*	*Procamallanus *(*S.*)* rarus*	*Procamallanus *(*S.*)* pimelodus*	*Dadaytrema oxycephala*	*Brasilnema sp.*	Cystidicolidae gen. sp.	*Rhadochona acuminata*	
Prevalence	13.33 (0.16)	15.00 (0.25)	0.83 (0.0)	12.50 (0.02)	4.17 (0.01)	4.17 (0.0)	1.67 (0.0)	0.83 (0.0)	
Mean abundance	3.38 ± 11.29	3.85 ± 12.73	0.01 ± 0.03	0.57 ± 1.73	0.08 ± 0.31	0.04 ± 0.15	0.02 ± 0.06	0.02 ± 0.07	
Mean intensity	25.31 ± 20.21	25.67 ± 30.66	1.00 ± 0.13	4.53 ± 2.94	2.00 ± 0.81	1.00 ± 0.28	1.00 ± 0.18	2.00 ± 0.26	
Total abundance	405	462	1	68	10	5	2	2	
Parameters	Species								
Rainy season	*Orientatractis moraveci*	*Rondonia rondoni*	*Cucullanus pinai pinai*	*Procamallanus *(*S.*)* pimelodus*	*Dadaytrema oxycephala*	*Brasilnema* sp*.*	*Rhadochona acuminata*		
Prevalence	10.00 (0.29)	4.17 (0.24)	1.67 (0.01)	23.33 (0.04)	1.67 (0.0)	10.83 (0.10)	1.67 (0.0)		
Mean abundance	3.53 ± 12.22	1.97 ± 1.97	0.08 ± 0.32	1.23 ± 2.90	0.03 ± 0.10	2.53 ± 7.94	0.02 ± 0.06		
Mean intensity	35.33 ± 35.86	47.20 ± 29.16	5.00 ± 1.06	5.25 ± 4.88	1.50 ± 0.29	23.38 ± 12.88	1.00 ± 0.18		
Total abundance	424	236	10	147	3	304	2		

Concerning prevalence, *O. moraveci* had higher prevalence rates in the Xapuri River, although marginally significant (*χ*^2^ = 3.309, *p* = 0.069). *R. rondoni*, *P.* (*S.*) *pimelodus*, and *Brasilnema* sp. prevalences were significantly higher in the Xapuri River (*χ*^2^ = 7.067, *p* = 0.008; *χ*^2^ = 17.82, *p* = 0.00; *χ*^2^ = 9.808, *p* = 0.002, respectively). *P. acreanensis* occurred only in the Acre River, while *P.* (*S.*) *rarus* and Cystidicolidae gen. sp. occurred only in the Xapuri River. The other species did not have significant differences in prevalence between rivers.

In relation to season, *O. moraveci* had significant higher prevalence in the dry season only in the Acre River (Acre: *χ*^2^ = 5.128, *p* = 0.024; Xapuri: *χ*^2^ = 0.647, *p* = 0.421). *R. rondoni* had significantly higher prevalence in the dry season only in the Xapuri River (Acre: *χ*^2^ = 0.115, *p* = 0.734; Xapuri: *χ*^2^ = 8.127, *p* = 0.004). On the other hand, *P. pimelodus* had higher prevalence in the rainy season, also in the Xapuri River (Acre: *χ*^2^ = 1.778, *p* = 0.182; Xapuri: *χ*^2^ = 4.788, *p* = 0.029). In the Acre River, *P. acreanensis* was found only in the dry season and *Brasilnema* sp. only in the rainy season and the other species did not show significant differences between seasons. For the Xapuri River, *C. *(*C.*)* pinnai pinnai* and *Brasilnema* sp. occurred only in the rainy season, while *P. *(*S.*)* rarus *and Cystidicolidae gen. sp. only in the dry season. The other species did not show significant differences.

The GLM analysis carried out for *O. moraveci* abundance in relation to river and season indicated the null model as one of the plausible ones; however, the model which included river was also plausible (Table 3), as this species had higher prevalence, abundance, and intensity in the Xapuri than in the Acre River (Tables 1 and 2). Abundances of *R. rondoni*, *P. *(*S.*)* pimelodus*, and *Brasilnema* sp. were considerably higher in the Xapuri River (Tables 1 and 2). The models which included river and season were the most plausible ones, although for *R. rondoni*, the null model was also plausible (Table 3). *R. rondoni* abundances were higher in the dry season, while *P.* (*S.*) *pimelodus* and *Brasilnema* sp. abundances were higher in the rainy season (Tables 1 and 2).

**Table 3:** Generalized linear models (GLMs) of the effects of location (Acre and Xapuri Rivers) and season (rainy and dry) on the helminth abundance of *Pimelodus blochii*, in Acre and Xapuri Rivers, State of Acre, Brazil. Plausible models (∆AICc < 2) are in italics

	AICc	∆ AICc	w AICc
*Orientatractis moraveci*			
*Null*	*2200.6*	*0*	*0,44*
*River*	*2201.4*	*0.8*	*0.29*
River + season	2203.4	2.8	0.11
Season	2202.6	2.0	0.16
*Rondonia rondoni*			
*River*	*2150.6*	*0*	*0.48*
*River + season*	*2152.0*	*1.4*	*0.24*
*Null*	*2152.4*	*1.8*	*0.19*
Season	2153.8	3.2	0.10
*Procamallanus *(*S.*)* pimelodus*			
*River + season*	*1281.90*	*0*	*0.76*
*River*	*1281.91*	*0.01*	*0.76*
Null	1285.60	3.70	0.12
Season	1285.60	3.70	0.12
*Brasilnema* sp.			
*River + season*	*1588.3*	*0*	*0.94*
Season	1594.8	6.50	0.04
River	1595.4	7.10	0.03
Null	1601,7	13,40	0,00

### Community structure

**Fig. 2:**
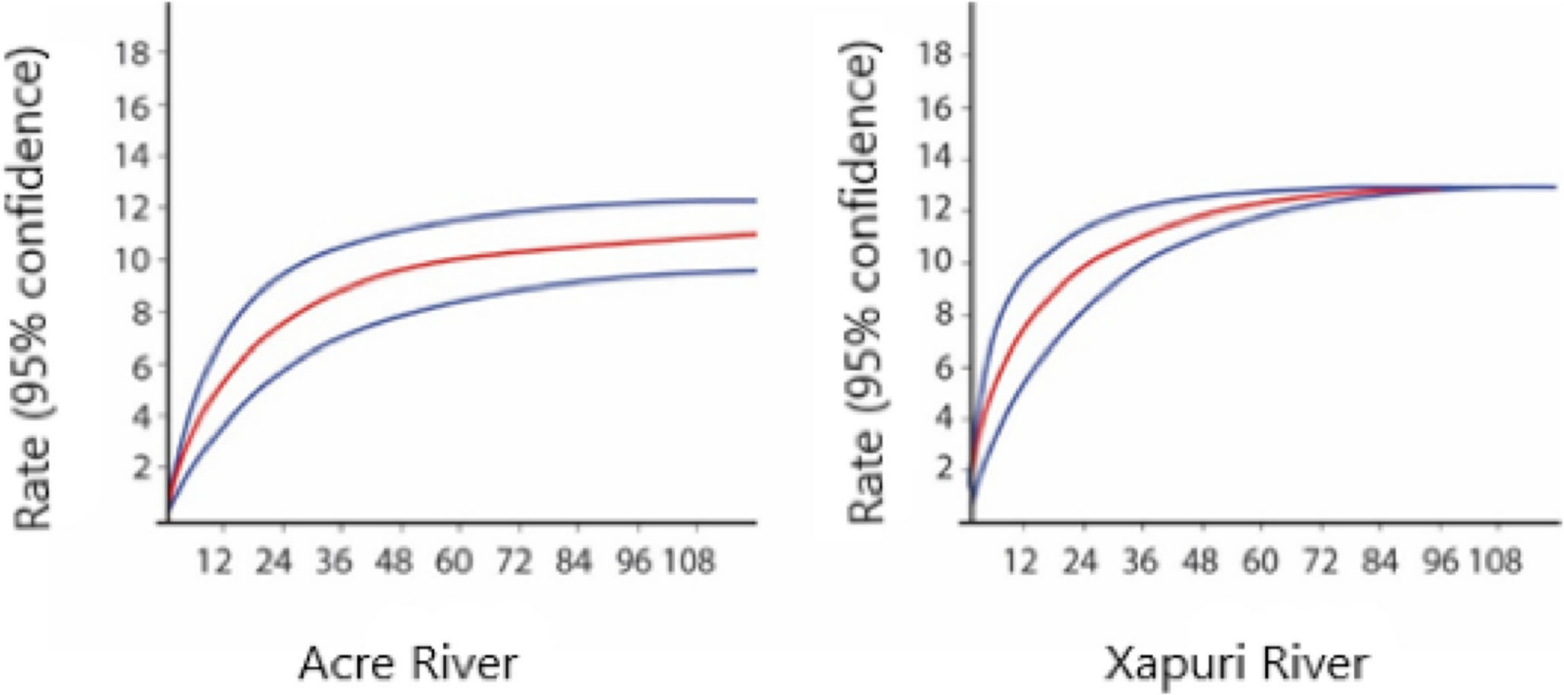
Sample-based rarefaction curves of helminth species richness (± 95 % CI) of *Pimelodus blochii*, in Acre and Xapuri Rivers, State of Acre, Brazil

The rarefaction curves of the helminth species stabilized for both rivers, with a trend for earlier stabilization in the Acre River (Fig. 2). Although the total helminth species richness did not differ greatly between rivers (Table 4), mean species richness in the Xapuri River was more than twice the Acre’s values (Table 4). Estimated species richness indicated that the sample size comprised the helminth species present in the study areas (Table 4). Moreover, species diversity, according to the Shannon Indices, was significantly higher in the Xapuri River (*t* = − 7.064, *p* = 0.000, *df* = 732) (Table 4), which also showed higher species equitability and less species dominance in relation to the Acre River (Table 4).

**Table 4:** Diversity indices of the helminth species of *Pimelodus blochii* in Acre and Xapuri Rivers, State of Acre, Brazil

Acre River	Dry season	Rainy season
Total richness	7	7
Chao-1 estimated richness	7	7
Shannon diversity index	0.933	0.987
Equitability index	0.479	0.507
Berger-Parker dominance index	0.715	0.694
Xapuri River	Dry season	Rainy season
Total richness	8	7
Chao-1 estimated richness	8	7
Shannon diversity index	1.011	1.384
Equitability index	0.486	0.711
Berger-Parker dominance index	0.483	0.376
Total	Acre River	Xapuri River
Total richness	8	9
Chao-1 estimated richness	8	9
Mean richness	0.51	1.06
Shannon diversity index	0.988	1.331
Equitability index	0.475	0.605
Berger-Parker dominance index	0.707	0.398

Among the 45 fish found parasitized in the Acre River, 69% were infected by one helminth species, 27% by two species, and 4% by three species. Among the 66 parasitized fish in the Xapuri River, 39.4% were infected by one helminth species, 39.4% by two species, 12.1% by three species, 7.6% by four species, and 1.5% by five species.

Differences between seasons were not observed in relation to total species richness (7 in each season), or diversity (*t* = − 0.597, *p* = 0.551, *df* = 466) in the Acre River (Table 4). In addition, seasonal differences in mean species richness were very small (rainy = 0.53; dry = 0.48). Equitability and dominance indices also showed little differences between seasons for this river (Table 4). Meantime, in Xapuri, there was higher species diversity in the rainy season (*t* = − 12.173, *p* = 0.000, *df* = 1713), and also a higher equitability when compared with the dry season (Table 4). Nevertheless, we observed one more species in the dry season and similar mean species richness between seasons (Table 4). During the dry season, few species showed higher abundances (*O. moraveci* and *R. rondoni*) in relation to the other species (Table 2), resulting in less equitability.

The helminth community in the Acre River contained three dominant species: *O. moraveci*, *R. rondoni*, and *P. *(*S.*)* pimelodus*; the other fiver species were co-dominants (Table 5). In the Xapuri River, four species were dominant, the same observed in the Acre River and *Brasilnema* sp., *C.* (*C.*) *pinai pinai*, *D. oxycephala*, and *R. acuminata* were considered co-dominants and the others were subordinate (Table 5). The larvae recovered were considered unsuccessful pioneers.

**Table 5:** Indices of importance of the helminth species of *Pimelodus blochii* in Acre and Xapuri Rivers, State of Acre, Brazil

	Index of importance	Classification
Acre River		
*Orientatractis moraveci*	78.67	Dominant
*Rondonia rondoni*	5.34	Dominant
*Philometroides acreanensis*	0.02	Co-dominant
*Cucullanus *(*C.*) *pinai pinai*	0.66	Co-dominant
*Procamallanus *(*S.*)* pimelodus*	14.18	Dominant
*Dadaytrema oxycephala*	0.59	Co-dominant
*Rhadochona acuminata*	0.10	Co-dominant
*Brasilnema* sp.	0.44	Co-dominant
*Hysterothylacium* sp.	0.00	Unsuccessful pioneer
*Anisakis* sp.	0.00	Unsuccessful pioneer
Nematoda. gen. sp.	0.00	Unsuccessful pioneer
Acanthocephala gen. sp.	0.00	Unsuccessful pioneer
Xapuri River		
*Orientatractis moraveci*	42.83	Dominant
*Rondonia rondoni*	29.62	Dominant
*Cucullanus *(*C.*)* pinai pinai*	0.04	Co-dominant
*Procamallanus *(*S.*)* rarus*	0.00	Subordinate
*Procamallanus *(*S.*)* pimelodus*	17.06	Dominant
*Dadaytrema oxycephala*	0.17	Co-dominant
*Brasilnema* sp.	10.26	Dominant
Cystidicolidae gen. sp*.*	0.01	Subordinate
*Rhadochona acuminata*	0.02	Co-dominant
*Hysterothylacium* sp.	0.00	Unsuccessful pioneer
*Contracaecum* sp.	0.00	Unsuccessful pioneer
*Anisakis* sp.	0.00	Unsuccessful pioneer
Nematoda*.* gen. sp.	0.00	Unsuccessful pioneer
Acanthocephala gen. sp.	0.00	Unsuccessful pioneer

The estimated overall beta-diversity was 0.556. When partitioning this beta-diversity into turnover and nestedness components, we observed that nestedness was responsible for most of the beta-diversity (*β*turn = 0.0033; *β*nes = 0.553).

## Discussion

*Pimelodus blochii* is a new host for the nematode genus *Brasilnema*. All the other helminths have already been reported for this host. Except for the trematode *D. oxycephala*, all the other adult specimens were nematodes. The nematodes are among the most abundant endohelminths in *Pimelodus* spp., as reported in the studies at Mogi Iguaçu River, São Paulo State (Kohn and Fernandes 1987), Itajaí-Açu River, Santa Catarina State (Bachmann et al. 2007), São Francisco River, Minas Gerais State (Sabas and Brasil-Sato 2014), and Iaco and Acre Rivers, Acre State (Negreiros et al. 2018), corroborating the results of the present study.

The high prevalence of *P.* (*S.*) *pimelodus* is a common pattern in *P. blochii*, which is occasionally parasitized by *P.* (*S.*) *rarus.* Sabas and Brasil-Sato (2014) reported a high prevalence of *P.* (*S.*) *pimelodus* corroborating this relevant pattern and showing the same structural composition of camallanid species in congeneric pimelodid hosts. As the genus *Pimelodus* (Lacepède) is considered a paraphyletic assembly that requires revision (Lundberg and Littmann 2003; Lundberg et al. 2011), the existence of three species of congeneric hosts, from very distinct regions, parasitized by the same species (*P.* (*S.*) *pimelodus*) may suggest genetic similarity of the hosts and/or low specificity of the parasite, justifying its wide geographical distribution.

As *P.* (*S.*) *pimelodus*, *O. moraveci* also stands out from the other species in the community, as it was the most prevalent and abundant species in the Acre River and the second most prevalent in the Xapuri River. These results differ from the study of Negreiros et al. (2018), which reported this species in low prevalence and abundance rates.

The pathogenicity of fish parasites is mostly associated with the species of the genera *Procamallanus* and *Cucullanus.* They may cause lesions and inflammatory reactions in the intestinal mucosa because of their mouth capsules for fixation and blood feeding habit, which may cause anemia in the hosts (Santos et al. 2013). In small fishes, they can lead to a growth impairment and cause intestinal obstruction. Regarding philometrid species, there are several records in freshwater environments in Brazil parasitizing cavities and body surfaces of fishes, but little is known about their pathogenic potential (Santos et al. 2013). Besides these two genera, *Philometroides acreanensis*, due to its considerable size, may also compromise the host health when it occurs in high prevalence. However, this species was found in only one fish caught in the Acre River. This helminth species was reported in this host with a prevalence of 3.8% in the same river by Negreiros et al. (2018).

Among nematodes *Contracaecum* sp., *Hysterothylacium* sp., and Anisakidae gen. sp. larvae, *P. blochii is* used as an intermediate host. Concerning the acanthocephalans, only small cysts were found in the abdominal cavity of the fish, showing their role as intermediate or paratenic hosts in the life cycle of these parasites. Besides, pimelodids have also been reported as definitive hosts as adults of *Neoechinorhynchus* (*Neoechinorhynchus*) *pimelodi* Brasil-Sato and Pavanelli 1998 were observed in *Pimelodus maculatus* (Lacépède 1803) from Três Marias reservoir (Brasil-Sato and Pavanelli 1998; Santos et al. 2008).

Helminth abundance and prevalence were widely influenced by seasonality and by the river in which the hosts were captured. Differences found between rivers were more evident for *R. rondoni*, *P.* (*S.*) *pimelodus*, and *Brasilnema* sp., which were more abundant and prevalent in the Xapuri River. In addition, the absence of *P.* (*S.*) *rarus* and Cystidicolidade gen. sp. in the Acre River may indicate that these species are more susceptible to the environmental local conditions where the hosts are living. The occurrence of only two female specimens of *P. acreanensis* only in the Acre River may indicate that possible intermediate hosts are restricted to the Acre River. Negreiros et al. (2018) also found few specimens of this helminth in *P. blochii* in the same river. Those authors also found more local environmental than seasonal influence in the Acre River, and seasonal differences were clearer in the most preserved river (Negreiros et al. 2018).

The higher prevalence of *P.* (*S.*) *pimelodus* observed during the rainy season in both rivers is in disagreement with the findings of Negreiros et al. (2018), which reported higher prevalence during the dry season. The authors argue that, during the dry season, the catfish becomes generalist in its dietary habit, what may increase the probability of ingesting intermediate hosts of this parasite. However, rainfall indices were higher during the years of the present study when compared with Negreiros et al.’s, what may have influenced the discrepant results.

*Rondonia rondoni*, which occurred predominantly during the dry season, is known to occur in different fishes, in different river systems, and in high intensities (Kohn et al. 1985; Moravec 1998). They are viviparous and its direct life cycle may allow the spread of numerous eggs, with several filaments, and larvae in the marginal vegetation of low water bodies during the dry season.

Considering the effects of seasonality on the helminth biodiversity, differences were more pronounced for Xapuri’s fishes, probably due to the larger helminth abundance in this area. The rainy season increases the river area where the fishes can occupy, which may favor increases in population sizes. It is known that the reproductive peaks and spawning periods in this species mostly occur during the wet season (Guerrero et al. 2009). These factors may result in an increment in parasite diversity in this host. In this case, a larger number of infracommunities, which is the parasite community of each individual host, would allow the occurrence of more species of parasites. Differences in helminth species composition and abundance over time were observed by Negreiros et al. (2019b), which attributed those differences to the natural variation in the aquatic environment of the Amazon region, influencing the host diet and susceptibility to parasites.

The importance indices together with the parasitological parameters and the beta-diversity analysis provide important information concerning the parasite community structure. Communities where the nestedness component has a larger contribution to the beta-diversity than the turnover component are expected to have a group of species that would be present in most of the communities, which can be considered as core species. In the same way, rare species occurring in only a few communities can be considered as satellite species. The importance indices indicate that *O. moraveci*, *R. rondoni*, and *P. *(*S.*)* pimelodus* form the main core species of the helminth communities of *P. blochii*, regardless of locality and season of the year. The results suggest that such species have wider tolerance limits along their environmental gradients (hosts and abiotic milieu) in relation to the others in the studied area. Notwithstanding, the highest prevalence observed (35.83% for *P.*(*S.*)* pimelodus* in the Xapuri River) also indicates that, even in those core species, few infrapopulations are infected by each species, corroborating the aggregated patters of distribution. The subordinate species found in Xapuri’s fishes (*P.* (*S.*) *rarus* and Cystidicolidae gen. sp.), which occurred only in this river, as well as the co-dominant species *P. acreanensis*, registered only in Acre’s fishes, represent satellite species forming other subsets on the helminth communities.

These helminth community structures corroborated the results of the beta-diversity, which indicated that the contribution of the nestedness component was much larger than of the turnover component for the beta-diversity pattern observed. Beta-diversity was firstly proposed by Whittaker (1960) and became commonly used to analyze the relationship between the spatial structures of species with the ecological processes (Ricotta 2017). In addition, communities can be compared in relation to species composition using dissimilarities indices (Legendre and Legendre 1998). The turnover component of the beta-diversity indicates that the differences between communities occur by the substitution of species from one community to another, whereas the nestedness component implies in loss of species when comparing communities, indicating that a community presents species that form a subset of another (Baselga 2010). Thus, our results indicate that the helminth community from Acre’s fishes forms a subset of the helminth community of the Xapuri’s, considering not only the species composition but also the species diversity between localities. Moreover, in Acre, infracommunities had a maximum of three species, while in Xapuri, we found a few infracommunities with four or five helminth species. Yet, the significant difference observed in the diversity indices and the higher mean species richness between rivers also reinforces the fact that the helminth community of *P. blochii* in the Xapuri River has higher species diversity when compared with that in the Acre River.

This is the first study of the parasite community of *P. blochii* in the Xapuri River. We suggest that the anthropic action related to the damping of human sewage in the Acre River may be affecting the structure of the helminth community of *P. blochii* in this area and thus reducing its species diversity when compared with the Xapuri River, which is considered a preserved river in terms of water quality. The results observed are in agreement with our hypothesis that local characteristics of the environment may influence the helminth community diversity.
